# Does the Genotype Have a Significant Effect on the Formation of Intra-Annual Density Fluctuations? A Case Study Using *Larix decidua* from Northern Poland

**DOI:** 10.3389/fpls.2016.00691

**Published:** 2016-05-20

**Authors:** Marcin Klisz, Marcin Koprowski, Joanna Ukalska, Cristina Nabais

**Affiliations:** ^1^Department of Silviculture and Genetics, Forest Research Institute in PolandSekocin Stary, Poland; ^2^Department of Ecology and Biogeography, Faculty of Biology and Environmental Protection, Nicolaus Copernicus UniversityToruń, Poland; ^3^Biometry Division, Department of Econometrics and Statistics, Faculty of Applied Informatics and Mathematics, Warsaw University of Life SciencesWarsaw, Poland; ^4^Department of Life Sciences, Centre for Functional Ecology, University of CoimbraCoimbra, Portugal

**Keywords:** IADF, genetics, G × E, European larch, generalized estimating equations, wood density

## Abstract

Intra-annual density fluctuations (IADFs) can imprint environmental conditions within the growing season and most of the research on IADFs has been focused on their climatic signal. However, to our knowledge, the genetic influence on the frequency and type of IADFs has not been evaluated. To understand if the genotype can affect the formation of IADFs we have used a common garden experiment using eight families of *Larix decidua* established in two neighboring forest stands in northern Poland. Four types of IADFs were identified using X-ray density profiles: latewood-like cells within earlywood (IADF-type E), latewood-like cells in the transition from early- to latewood (IADF type E+), earlywood-like cells within latewood (IADF-type L), and earlywood-like cells in the border zone between the previous and present annual ring (IADF-type L+). The influence of explanatory variables i.e., families, sites, and years on identified density fluctuations was analyzed using generalized estimating equations (GEE). We hypothesized that trees from different families will differ in terms of frequency and type of IADFs because each family will react to precipitation and temperature in a different way, depending on the origin of those trees. The most frequent fluctuation was E+ and L types on both sites. The most important factors in the formation of IADFs were the site and year, the last one reflecting the variable climatic conditions, with no significant effect of the family. However, the relation between the formation of IADFs and selected climate parameters was different between families. Although, our results did not give a significant effect of the genotype on the formation of IADFs, the different sensitivity to climatic parameters among different families indicate that there is a genetic influence.

## Introduction

Radial growth of trees reflects the interactions between external (environmental) and internal factors (physiological processes determined in relation to genotype; Savva et al., 2002). The influence of environment (mainly climatic) has been very well-documented in numerous studies (Wimmer et al., 2000; Rigling et al., 2001; Masiokas and Villalba, 2004). Much work has also addressed the influence that biotic and abiotic environmental variables exert in generating anomalies in the course of xylogenesis (Dmuchowski et al., 1997; Eilmann et al., 2013; Vieira et al., 2015).

Trees' plasticity expressed in terms of stress-induced growth reactions is known to differ in line with the expression of the genotype determining the physiological processes (López-Maury et al., 2008). Differences in the radial reaction are most probably dependent on trees' degrees of relatedness at the level of the species, provenance, or genotype. The few long-term provenance experiments that have been carried out confirm inter-population variability of the radial reaction as expressed in terms of tree ring width (McLane et al., 2011; Kalliokoski et al., 2012; Wilczyński and Kulej, 2013).

The temperature increase and precipitation anomalies observed in recent years, assuming the form of droughts, heatwaves, and heavy rainfall (Lindner et al., 2014), also finds reflection in the anatomical structure of annual rings (Masiokas and Villalba, 2004; Rozas et al., 2011; Seo et al., 2011). The typical anatomical structure, with a clear distinction between early- and latewood, can be modified in a growing season with climatic conditions considered non-typical (Wimmer, 2002). In this sense, latewood-like cells can appear within earlywood, accounted for as a physiological reaction of plants to stress associated with a shortage of water, to prevent risks of cell embolism (Battipaglia et al., 2014). On the other hand, at the end of a growing season, which has featured a water shortage, the arrival of precipitation can lead to the appearance of modifications in the anatomical structure of latewood cells, with the formation of earlywood-like cells (Vieira et al., 2015). These types of modifications are classified as intra-annual density fluctuations (IADFs). Campelo et al. (2013) proposed a classification of four types of IADFs based on their location within the tree-ring: IADF-type E, with latewood-like cells within earlywood; IADF-type E+, representing a smooth transition between early- and latewood cells; IADF-type L, with earlywood-like cells within latewood; and IADFs-type L+, with earlywood-like cells between the end of the latewood and the earlywood of the next tree ring. This classification for wood density fluctuations has been used for the purposes of the work described here.

Previous attempts to explain differences in trees' reactions to climatic anomalies, as manifested in the development of density fluctuations in wood, have concentrated on the aspect of the variability characterizing trees differing in terms of size or age (Vieira et al., 2009; Campelo et al., 2013, 2015; Novak et al., 2013), or aspects reflecting intraspecific competition (biosocial position or growth rate; Copenheaver et al., 2006). Differences in the radial-growth reaction relating to soil-moisture conditions have also been investigated (Battipaglia et al., 2010; de Luis et al., 2011). Schweingruber (1980) emphasized that wide rings have more fluctuations than narrow ones. According to this, we can assume that climate conditions favoring growth can also affect the frequency of density fluctuations. The use of IADFs for environmental studies in different parts of Europe were published recently by Battipaglia et al. (2016). However, few works have taken into account the genotype effect on the formation of IADFs. It is known that the wood density of annual rings and its component elements (i.e., density of earlywood and latewood) are strongly determined genetically, with a high degree of heritability (Pâques, 2004; Fujimoto et al., 2008; Klisz, 2011). In conditions extremely unfavorable to growth (a water shortage induced by drought and high temperatures), it is possible to observe a high level of genetic control of the density profiles of annual rings, including the location of peak density within earlywood or the transition zone (Rozenberg et al., 2002). This findings support the idea that the frequency of occurrence of the different types of density fluctuation in wood might also be determined by a genetic factor.

The aim of this work was to determine the influence of the origin of trees on the frequency of different types of IADFs. We have used a common garden experiment (seed orchard) using half-sib families of *Larix decidua* established in two neighboring forest stands in northern Poland since 1985. We hypothesized that trees from different families will differ in terms of frequency and type of IADFs because each family will react to precipitation and temperature in a different way, depending on the origin of those trees.

## Materials and methods

### Study site and samples

**Figure 1:**
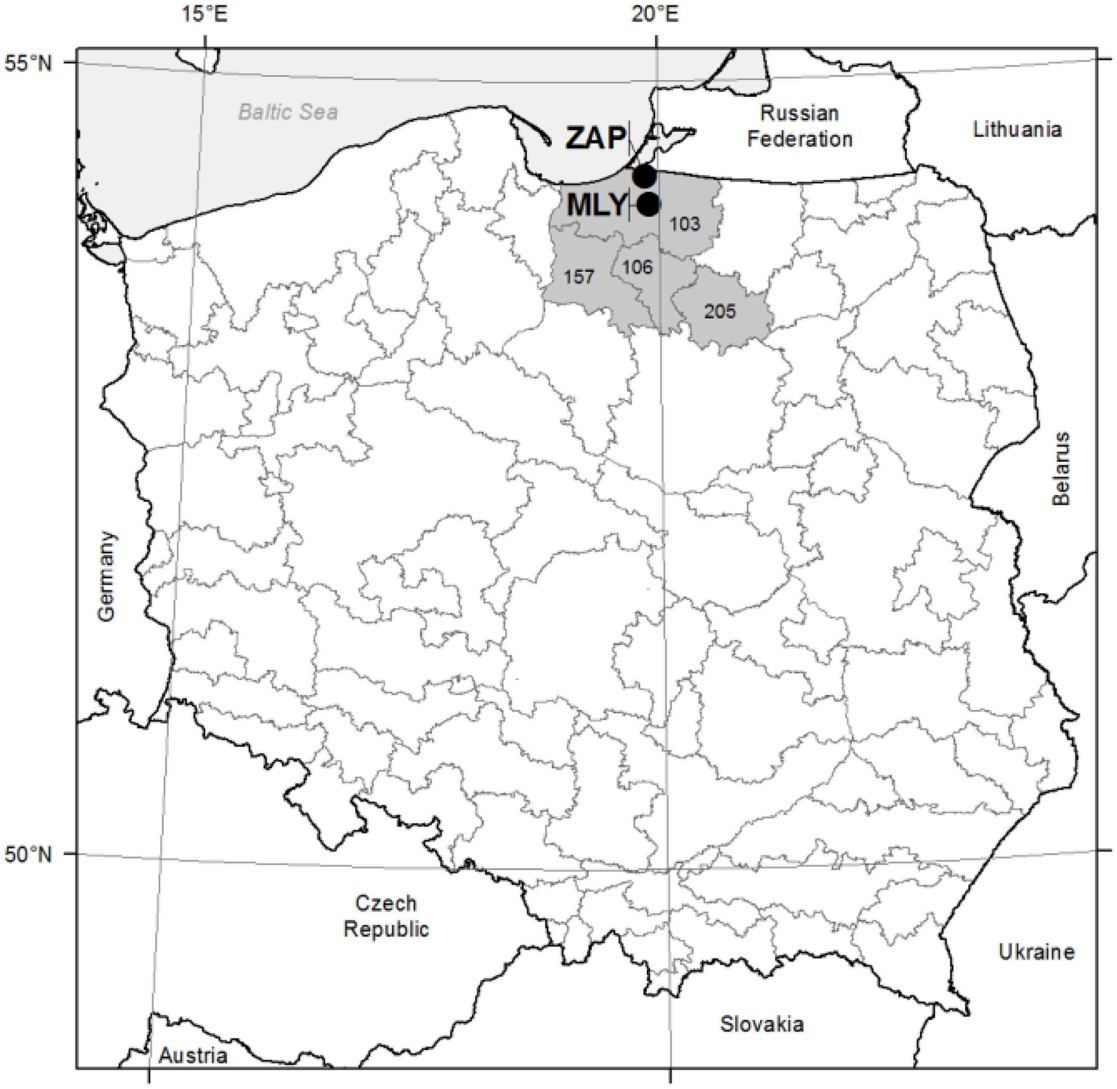
**Study sites location**. MLY—Młynary experimental site; ZAP—Zaporowo experimental site; 103, 106, 157, 205—seed regions. Gray line—seed regions boarders.

Two study areas (even-aged seed orchards) were established in 1985 in northern Poland, within the Gdańsk Coast (Pobrzeże Gdańskie) Macroregion, under similar site conditions within the Forest Districts of Młynary (fresh broadleaved forest; N: 54°13′E: 19°54′; 55 m a.s.l.) and Zaporowo (fresh mixed/broadleaved forest; N: 54°24′E: 19°51′; 10 m a.s.l.). The climate is temperate with a mean temperature of 7.6°C and annual precipitation of 742 mm, mostly distributed in June–November [climate data from European Climate Assessment & Dataset (ECA&D) project, weather station in Elblag and Kaliningrad, reference period 1948–2014; (Klein Tank et al., 2002)]. Site condition characterized sandy clay soil in Mlynary and brown earth soil in Zaporowo. Both of the seed orchards were laid out in line with a randomized complete block design represented by the progeny of 33 plus trees of European larch. The 33 families in turn represented four seed zones for the European larch in northern Poland. i.e., nos. 103, 106, 157, and 205 (Figure 1). Twenty-five families of European larch were represented by at least 20 specimens at the two experimental sites. Based on the microdensity profiles of these 25 families, eight wood density classes were established. Afterwards, a selection of eight families was made to study the frequency of the different types of IADFs (Klisz, 2011). The criteria to select eight families among the 25 were based on the representation of each wood density class. At each experimental site (seed orchard), at least 20 sample trees representative of each of the eight families of European larch were selected. A total of 188 trees were selected at the Młynary site, and 201 at Zaporowo. In 2007, by means of Pressler borer, two cores of 5 mm diameter were taken from each tree at a height of 1.3 m above the ground, from the eastern and southern directions.

### Wood properties

Each wood sample was subject to the standard preparatory procedure for X-ray scanning. Uniformity of wood-sample width was achieved by way of lengthwise cuts using two circular saws (Larsson et al., 1994). Resin extraction was carried out in distilled water (Grabner et al., 2005), while stabilization of sample humidity levels at 15% was achieved in the Itrax Scanner density scanner. To obtain each of the required microdensity profiles, samples of wood were X-ray scanned using the Itrax Scanner at constant parameters: voltage 35 V, exposure 50 ms and resolution 25 μm. The exact procedure for X-ray scanning of wood samples using an Itrax Density Scanner (Cox Analytical Systems) is described in Lindeberg (2004), Bergsten et al. (2001), and Fries and Ericsson (2006). The images obtained were analyzed using the WinDENDRO 2009b program to establish the density profiles. To define the limits of earlywood and latewood, a constant value of 70% of the maximum density of wood in the given annual ring was adopted. This was the value used in comparisons with sample material—for European larch, and with samples of Scots pine (constant value for 50% of maximum wood density in a given annual ring), as analyzed using the same method at the same laboratory, by Fries and Ericsson (2009). The tree-rings analyzed covered the period from 1990 to 2006. All sample trees had the same cambial age.

### Determination of different types of IADF

**Figure 2:**
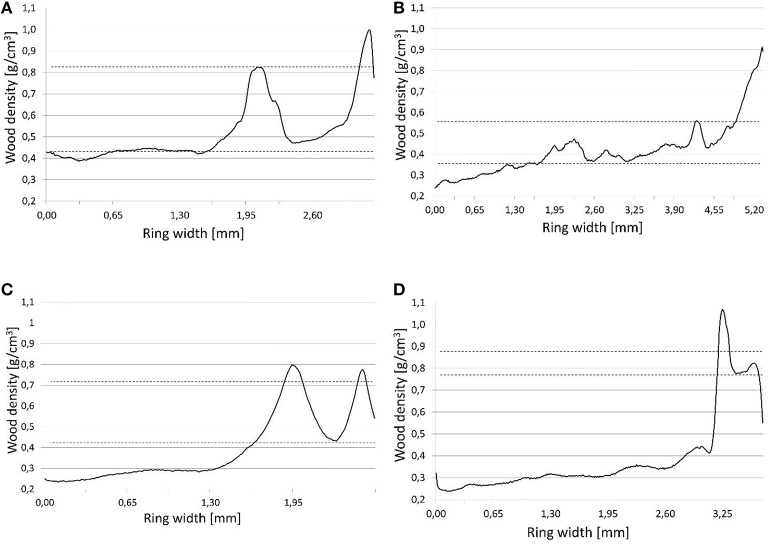
**IADFs types determination based on microdensity profiles**. **(A)** IADF E, **(B)** IADF E+, **(C)** IADF L, **(D)** IADF L+. Graphs **(A)** and **(B)**: Upper dashed line—IADFs maximum density, lower dashed line—average earlywood density. Graphs **(C)** and **(D)**: Upper dashed line—average latewood density, lower dashed line—IADFs minimum density.

In line with the locations of density fluctuations in annual rings, Campelo et al. (2013) identified four types of fluctuations. Our study improves on this classification, incorporating measured values for wood density. As a limit value allowing the occurrence of fluctuations to be identified, we adopted a microdensity local maximum >0.1 g/cm^3^ of the average early or latewood (Figure 2). The classification was then into E—high values for density in earlywood; E+—high values for wood density in the transition zone between earlywood and latewood; L—low values for density in latewood; and L+—low values for density in the latewood adjacent to the next annual ring. Determination of IADF occurrence was done for each core separately, and then the frequency value (present, absence) for each pair of cores was verified in order to avoid data duplication.

### Statistical analysis

#### The impact of the environment and genotype on IADF frequency

To determine the influence of provenances of the studied trees, site conditions, and years (the quality variables) on the presence of different types of IADF (the binary presence-absence dependent variable) we have used generalized estimating equations methodology (GEE). Proposed by Liang and Zeger (1986) as an extension of GzLM generalized linear models (Nelder and Weddenburn, 1972), this allows for the analysis of correlated data (Stokes et al., 2000). Use is made of a model assuming the form:

$$g(\mu_{ijk})=S_{i}+F_{j}+S\times F_{ij}+Y_{k}$$

where *g*(μ_*ijk*_) is the logit link function, μ_*ijk*_ is the mean for the *ij*th site × family combination in the *k*th year, S_*i*_ is the effect of the ith site (*i* = 1, 2), *F*_*j*_ is the effect of the *j*th family (*j* = 1, …, 8), *S* × *F*_*ij*_ is site × family interaction effect, and *Y*_*k*_ is the *k*th repeated measures year effect (*k* = 1, …, 17). The significance of model effects was tested using Wald statistics for the type 3 analysis. Decomposition of variance (DOV) was performed to further quantify how much variance in the predicted IADF can be attributed to site, family, site × family interaction, and year effects (Huang et al., 2014). The contrast analysis was in turn applied for comprehensive comparisons of significant model effects. Differences between tested families in the IADFs frequencies were shown on diffograms (mean-mean scatter plot). The analyses were carried out using SAS 9.3 software (SAS Institute Inc, 2011), and the GENMOD procedure (Stokes et al., 2000) was followed.

#### Relationships between climate and IADF

To investigate climate-IADF relationships, a GzLM model was applied using the MASS package (Author: Brian Ripley) from R (R Development Core Team, 2007). Climate data from two meteorological stations (Elblag and Kaliningrad) were obtained from the European Climate Assessment & Dataset (ECA&D) project (Klein Tank et al., 2002). For the purposes of the study, a dependent relationship between earlywood and climate data from March to June was assumed, as well as between latewood and the climate between May and September. The most parsimonious model according to AIC criterion was applied to the different IADF types, for each family and site (Akaike, 1974). A chi-square test for the type 3 analyses was applied to check for the significance of relationships. On the basis of Wald estimates a percent change in the odds of the occurrence of IADF for each 1-unit increase of climatic variables was determined (Allison, 2012).

## Results

### IADF frequency

At the Młynary and Zaporowo site, 9.1 and 11.1% of all rings showed the occurrence of IADFs, respectively (Table 1). Taking into account all tree-rings with IADFs, the dominant type at both experimental sites was type E+ accounting for 44.8% in the case of Młynary, and 42.6% in the case of Zaporowo. The second most frequently identified fluctuation at both sites was type L (35.8% in Młynary and 31.8% in Zaporowo). Taken together, the remaining two types of IADFs (E and L+) accounted for just 19.2% of fluctuations in trees from Młynary, and 26% of those recorded in trees from Zaporowo (Table 1).

**Table 1:** **Descriptive statistic of the intra-annual density fluctuations of *Larix decidua* from different families and growing in two sites in Northern Poland**.

**Family**	**No. of trees (cores)**		**Cores with IADFs**		**No. rings analyzed**		**%IADFs (No. rings)**		**%IADFs-E (No. rings)**		**%IADFs-E+ (No. rings)**		**%IADFs-L (No. rings)**		**%IADFs-L+ (No. rings)**	
	**Młynary**	**Zaporowo**	**Młynary**	**Zaporowo**	**Młynary**	**Zaporowo**	**Młynary**	**Zaporowo**	**Młynary**	**Zaporowo**	**Młynary**	**Zaporowo**	**Młynary**	**Zaporowo**	**Młynary**	**Zaporowo**
2514	21 (37)	25 (46)	25	33	563	618	8.3 (47)	9.9 (61)	14.9 (7)	32.8 (20)	38.3 (18)	42.6 (26)	36.2 (17)	23.0 (14)	10.6 (5)	1.6 (1)
2549	29 (47)	26 (42)	39	30	629	482	11.3 (71)	12.0 (58)	14.1 (10)	12.1 (7)	38.0 (27)	60.3 (35)	45.1 (32)	22.4 (13)	2.8 (2)	5.2 (3)
2817	30 (51)	27 (42)	38	36	767	478	9.0 (69)	14.0 (67)	15.9 (11)	26.9 (18)	47.8 (33)	44.8 (30)	36.2 (25)	26.9 (18)	0.0 (0)	1.5 (1)
2818	25 (45)	22 (33)	31	12	637	373	8.0 (51)	4.6 (17)	9.8 (5)	23.5 (4)	60.8 (31)	23.5 (4)	27.5 (14)	52.9 (9)	2.0 (1)	0.0 (0)
2860	20 (26)	25 (43)	19	40	374	577	13.4 (50)	12.1 (70)	14.0 (7)	22.9 (16)	40.0 (20)	41.4 (29)	38.0 (19)	34.3 (24)	8.0 (4)	1.4 (1)
2864	27 (42)	26 (42)	29	33	590	564	7.8 (46)	11.9 (67)	21.7 (10)	23.9 (16)	37.0 (17)	34.3 (23)	28.3 (13)	38.8 (26)	13.0 (6)	3.0 (2)
3076	19 (31)	21 (29)	24	25	429	379	8.9 (38)	12.7 (48)	10.5 (4)	22.9 (11)	52.6 (20)	33.3 (16)	31.6 (12)	41.7 (20)	5.3 (2)	2.1 (1)
3078	19 (35)	28 (53)	25	42	511	723	7.4 (38)	10.7 (77)	7.9 (3)	20.8 (16)	47.4 (18)	45.5 (35)	39.5 (15)	31.2 (24)	5.3 (2)	2.6 (2)
Total	190 (314)	200 (330)	230	251	4500	4194	9.1 (410)	11.1 (465)	13.9 (57)	23.2 (108)	44.9 (184)	42.6 (198)	35.9 (147)	31.8 (148)	5.4 (22)	2.4 (11)

**Figure 3:**
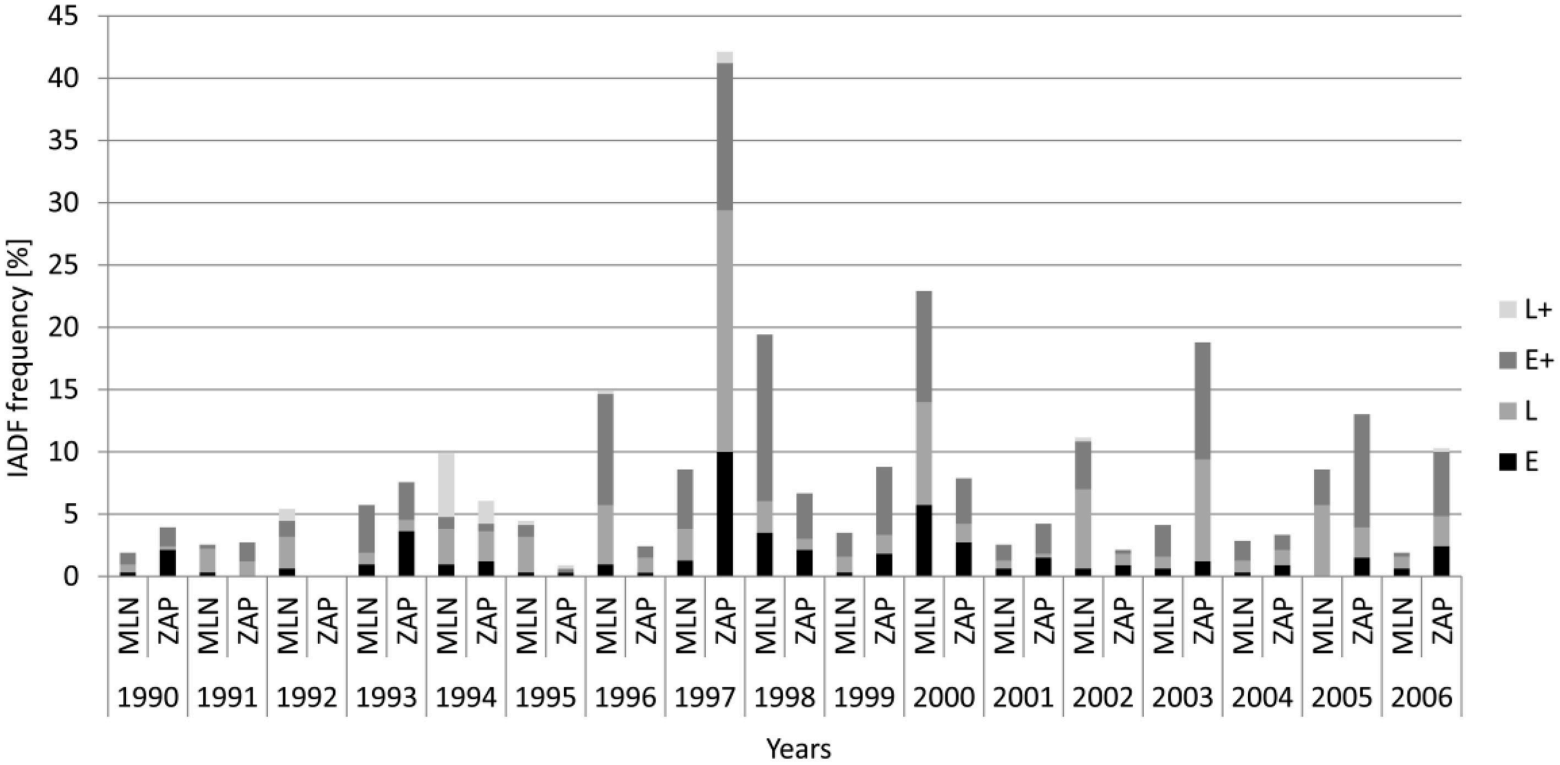
**Frequency of the different types of IADFs in relation to calendar years in the Młynary (MLN) and Zaporowo (ZAP) Forest Districts**.

From 1990 to 2006, trees at the Młynary site showed IADFs every year, while in Zaporowo no IADFs were observed in 1992 (Figure 3). At the Młynary site, the highest frequencies occurred in 1996, 1998, and 2000, with a high percentage of IADF type E+, accounting for 59.6, 68.9, and 38.9%, respectively (Figure 3). In turn, the highest frequency of IADFs observed in Zaporowo occurred in 1997 and 2003 dominated by IADF type L and type E+, respectively (Figure 3). At Młynary the highest frequencies of IADFs type L occurred in the years 1991, 1992, 1995, 2002, 2005, and 2006, while at Zaporowo, occurred in 1994, 1996, 1997, 2002, and 2004. The IADFs type L+ occurred in 1992 and 1994 at the Młynary, and in 1994 at Zaporowo.

### IADFs vs. site, family, and year

**Figure 4:**
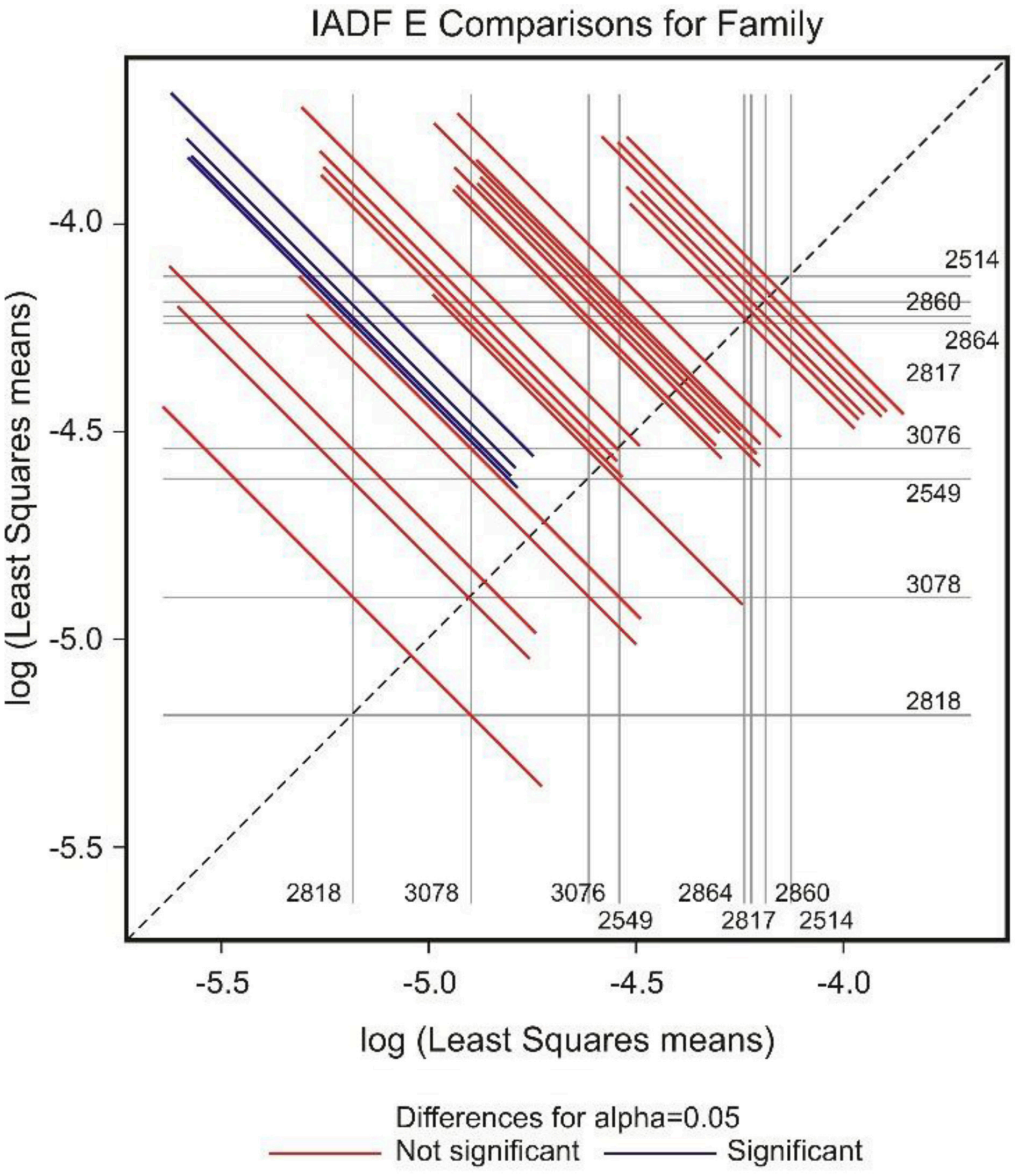
**Diffogram of differences in the IADF-type E frequencies between the European larch families**. The point of intersection of the horizontal and vertical line emanating from both axes for the two families represents the value of their difference (in log scale) and segments on both sides of the crossing point show the confidence interval for this difference. The difference between pair of families is significant when lower and upper endpoints of the confidence interval are either positive or negative i.e., they both are above or below the dotted line of equality.

GEE analysis allowed the identification of the significant factors (site, family, year, and their interactions) determining the frequency of the different types of IADFs. The most significant factors determining the formation of IADFs type E were the site and year, with *P* = 0.001 and *P* < 0.001, respectively (Table 2). Although, the families did not have a significant impact in the formation of IADFs type E (*P* = 0.099), it was possible to identify pairs of families that differed significantly in the frequency of IADFs type E. Family 2818 was found to differ significantly from families 2514, 2817, 2860, and 2864 (*P* = 0.018, 0.015, 0.014, and 0.021, respectively; Table 3, Figure 4).

**Table 2:** **Wald statistic for type 3 GEE analysis, the significance of the models' effects and the decomposition of variance DOV (%) for different IADFs' types**.

**Source of variation**	***DF***	**E-type**			**E+-type**			**L-type**			**L+-type**		
		**Chi-Square**	***P***	**DOV**	**Chi-Square**	***P***	**DOV**	**Chi-Square**	***P***	**DOV**	**Chi-Square**	***P***	**DOV**
Site	1	10.18	0.001	6.55	0.05	0.821	0.03	0.02	0.895	0.01	4.42	0.036	35.65
Family	7	12.05	0.099	7.76	13.34	0.064	7.96	8.26	0.311	4.25	7.98	0.334	64.35
Year	16	128.05	< 0.001	82.44	140.89	< 0.001	84.09	173.74	< 0.001	89.49			
Site × Family	7	5.05	0.654	3.25	13.27	0.066	7.92	12.12	0.097	6.24			

**Table 3:** **Significant differences between families and the different IADFs' types frequency**.

**IADF type**	**Family *i* vs. family *j***	**Differences of family LSM estimates (log scale)**	***P***	***OR***
E-type	2514 vs. 2818	1.057	0.018	2.877
	2817 vs. 2818	0.961	0.015	2.615
	2860 vs. 2818	0.944	0.014	2.701
	2864 vs. 2818	0.942	0.021	2.564
E+-type	2549 vs. 2818	0.890	0.008	2.436
	2817 vs. 2818	0.892	0.007	2.441
	2860 vs. 2818	0.958	0.005	2.608
	3076 vs. 2818	0.741	0.034	2.098
	3078 vs. 2818	0.711	0.031	2.036
	2860 vs. 2864	0.435	0.044	1.545
L-type	2860 vs. 2514	0.574	0.044	1.776
	2860 vs. 2818	0.799	0.013	2.222

P, p-value; OR, odds ratio. OR estimates are obtained by exponentiation of the differences of Family Least Squares Means (LSM) estimates. If OR > 1 Family i has OR higher odds of IADF presence than Family j.

**Figure 5:**
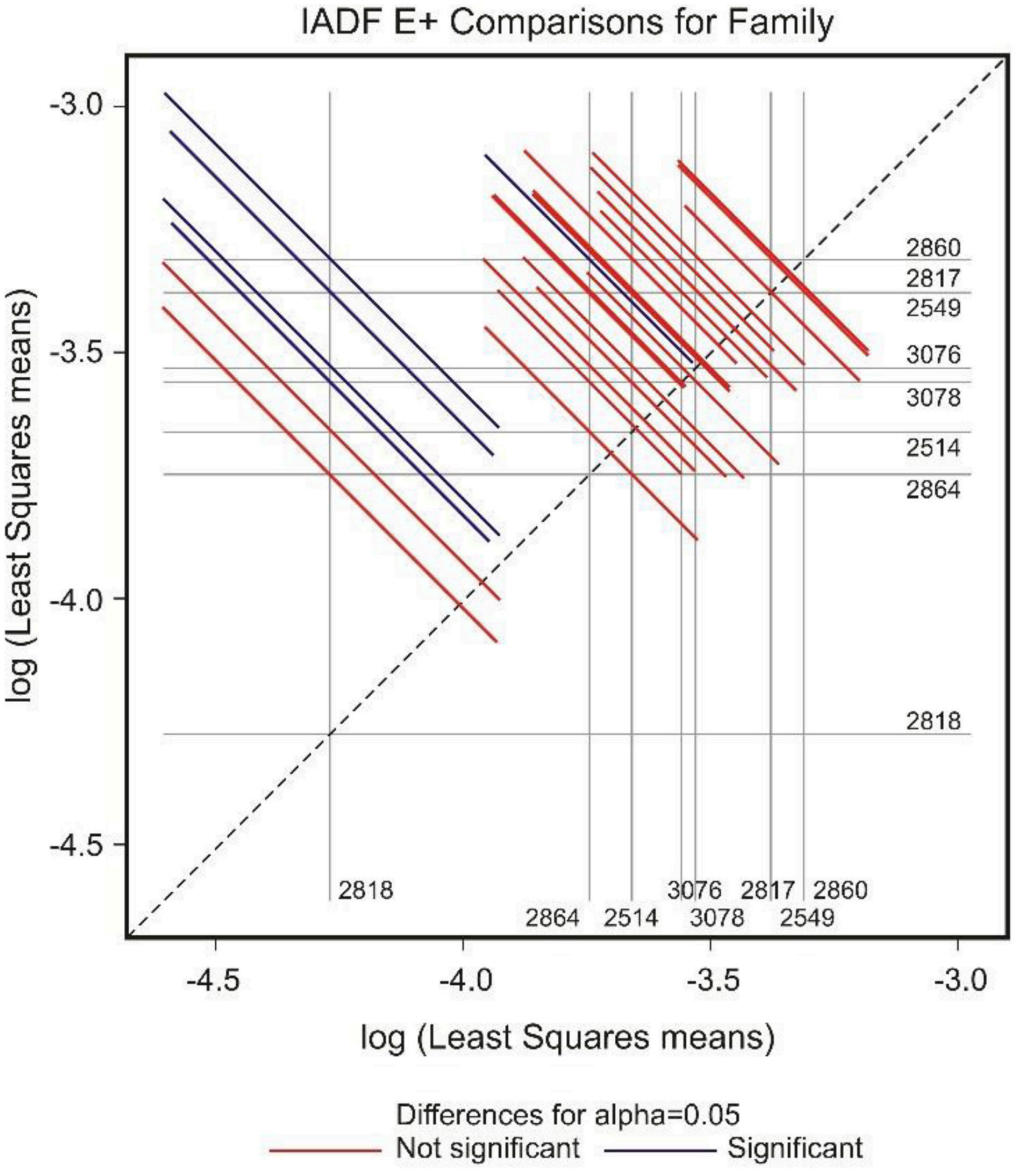
**Diffogram of differences in the IADF-type E+ frequencies between the European larch families**. The point of intersection of the horizontal and vertical line emanating from both axes for the two families represents the value of their difference (in log scale) and segments on both sides of the crossing point show the confidence interval for this difference. The difference between pair of families is significant when lower and upper endpoints of the confidence interval are either positive or negative i.e., they both are above or below the dotted line of equality.

In the case of IADFs type E+, the GEE analysis revealed that the only significant factor was associated with the calendar year (*P* < 0.001; Table 2). Nonetheless it was possible to distinguish pairs of families differing significantly in the frequency of IADFs type E+. Family 2818 showed a lower frequency of IADFs type E+ compared with 2549, 2817, 2860, 3076, and 3078 (Table 3, Figure 5). Additionally, family 2860 showed a higher frequency of IADFs type E+ compared with family 2864.

**Figure 6:**
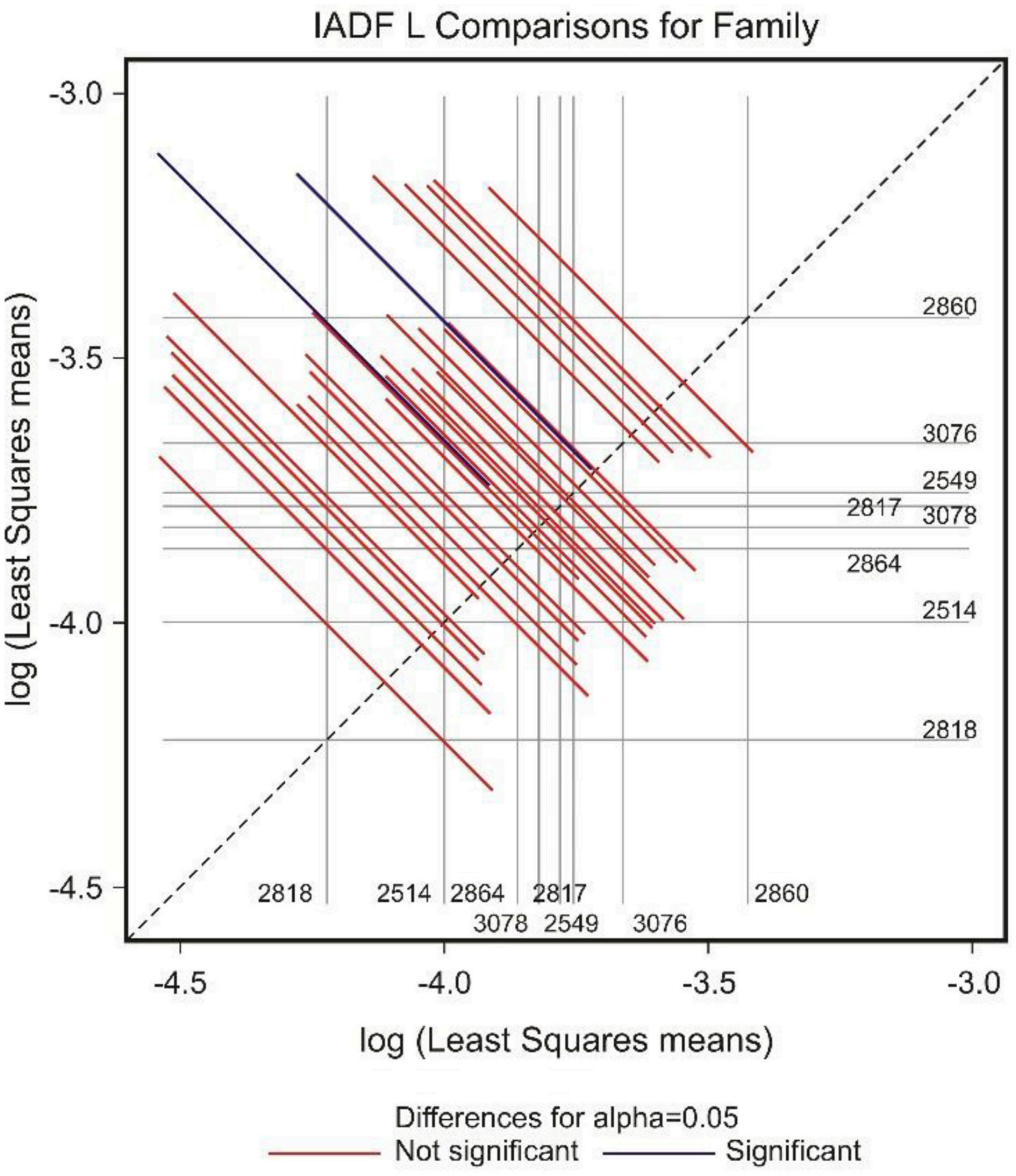
**Diffogram of differences in the IADF-type L frequencies between the European larch families**. The point of intersection of the horizontal and vertical line emanating from both axes for the two families represents the value of their difference (in log scale) and segments on both sides of the crossing point show the confidence interval for this difference. The difference between pair of families is significant when lower and upper endpoints of the confidence interval are either positive or negative i.e., they both are above or below the dotted line of equality.

The frequency of IADFs type L was significantly associated with the year (*P* < 0.001; Table 2). In the pairwise comparison of the different families, the family 2860 showed a higher frequency of IADFs type L compared with family 2818, and between family 2514 and 2860, it was marginally significant (Table 3, Figure 6).

The overall variation in IADFs frequency was strongly explained by the year effect, as suggested by a higher percentage of DOV, namely 82.4, 84.1, and 89.5% for IADFs type E, E+, and L, respectively. The site effect represents 6.55, 0.03, and 0.01%, and the family effect represents 7.76, 7.96, and 4.25% of the variation in the frequency of IADFs type E, E+, and L, respectively. Although, IADFs type L+ occurred in quite low frequencies, the overall variation in their frequency was explained by the family (64.4%) and site effect (35.7%).

**Figure 7:**
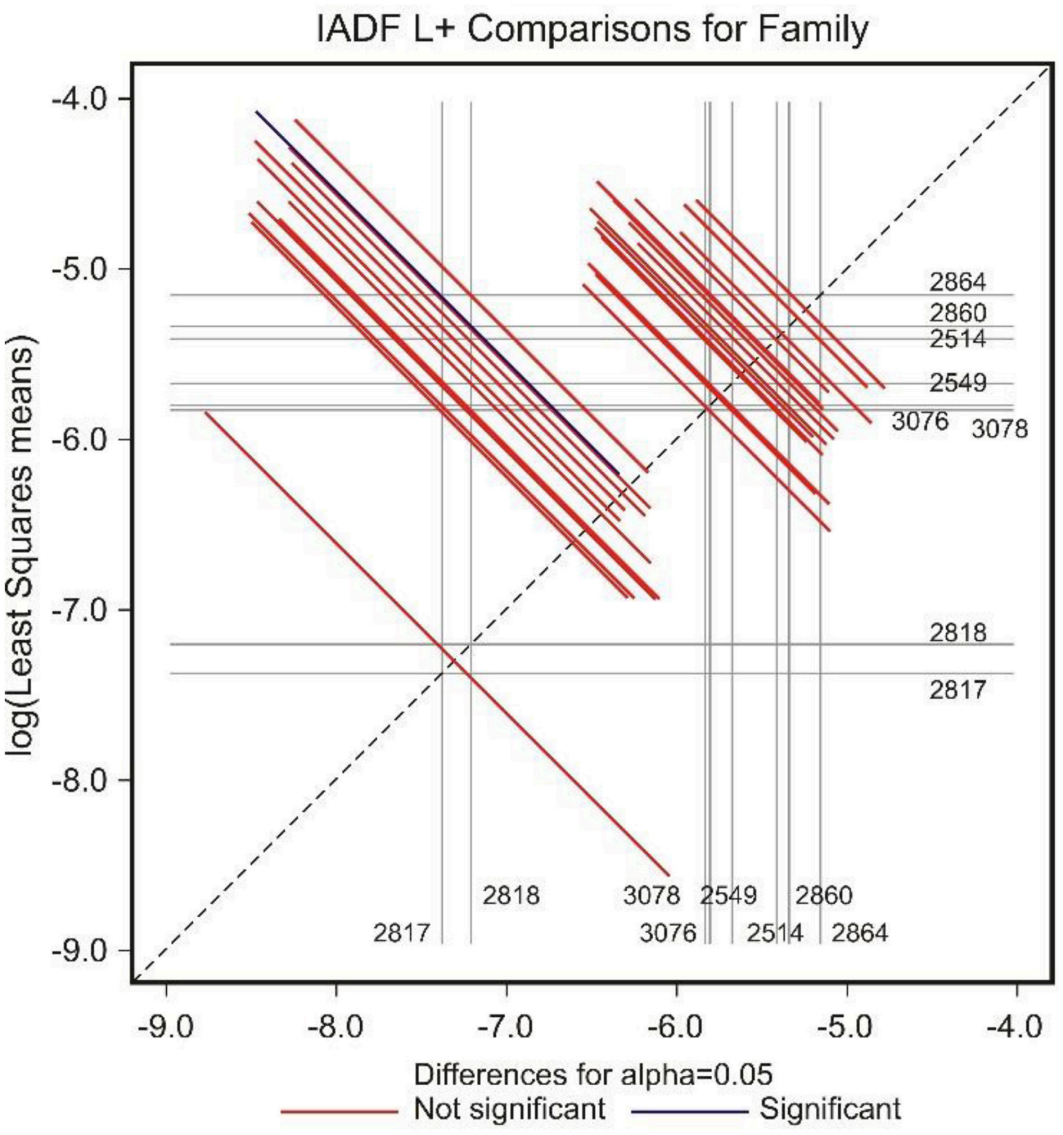
**Diffogram of differences in the IADF-type L+ frequencies between the European larch families**. The point of intersection of the horizontal and vertical line emanating from both axes for the two families represents the value of their difference (in log scale) and segments on both sides of the crossing point show the confidence interval for this difference. The difference between pair of families is significant when lower and upper endpoints of the confidence interval are either positive or negative i.e., they both are above or below the dotted line of equality.

The low number of observations of IADFs type L+ did not allow the factor year to be included in the model for the statistical analysis (Table 2). The site showed a marginal significance in the frequency of IADFs type L+. In the pairwise comparison of families, family 2864 showed a higher frequency when compared with family 2817 (Figure 7).

### IADFs and climate

In general, IADFs type E showed an increase with high precipitation in May and temperature in April, with more significant relationships occurring in Młynary, compared with Zaporowo (Table 4). IADFs type E of the family 2860 showed no climatic signal, in both sites. Concerning IADFs type E+ in general the most prominent significant relationships with climatic conditions were an increase of type E+ occurrence with high precipitation in March, April, and May (Table 5). IADFs type E+ also showed, in general, a decrease with temperature in March and an increase with temperature in April and May (Table 5). All families showed significant relationships with some of the climatic parameters. Interestingly, families 2864, 3076, 3078, 2860, and 2514 seem to respond earlier to climatic conditions, with an increase with precipitation in March and a decrease with the temperature also in March (Table 5). IADFs type E+ of the family 2860 growing in Zaporowo showed no significant climatic relationships.

**Table 4:** **Percent change (positive value indicates increase; negative value indicates decrease) in the odds of the occurrence of IADF type E for each 1-unit increase in monthly climatic data (mean temperature and total precipitation) from March to June, for the period 1990–2006**.

**Family**	**Site**	**Precipitation**				**Temperature**			
		**March**	**April**	**May**	**June**	**March**	**April**	**May**	**June**
2818	Młynary						15.67		
	Zaporowo		–9.8	4.55					
2817	Młynary			0.2					
	Zaporowo								
2549	Młynary						1.19		
	Zaporowo								
2864	Młynary	1.6		2.33					
	Zaporowo			0.07					
3076	Młynary								
	Zaporowo		0.05				–0.8	0.6	
3078	Młynary								
	Zaporowo								
2860	Młynary								
	Zaporowo								
2514	Młynary	2.7		4.49					
	Zaporowo			0.04					

Grayscale indicate the level of significance based on chi-square test—white p < 0.05, light gray p < 0.01, dark gray p < 0.001.

**Table 5:** **Percent change (positive value indicates increase; negative value indicates decrease) in the odds of the occurrence of IADF type E+ for each 1-unit increase in monthly climatic data (mean temperature and total precipitation), from March to June, for the period 1990–2006**.

**Family**	**Site**	**Precipitation**				**Temperature**			
		**March**	**April**	**May**	**June**	**March**	**April**	**May**	**June**
2818	Młynary		0.06		–0.03		0.63	0.72	
	Zaporowo						–14.72		
2817	Młynary			0.05				0.35	
	Zaporowo								
2549	Młynary		0.04					0.59	
	Zaporowo		0.02						
2864	Młynary						0.71		
	Zaporowo	0.04	0.07			–0.43	–0.82	0.59	
3076	Młynary			0.11			0.5		
	Zaporowo			–0.04		–0.37			
3078	Młynary	0.06		0.14			0.63		
	Zaporowo					–0.26			
2860	Młynary	0.07		0.1					
	Zaporowo								
2514	Młynary	0.03		0.053		–0.53			
	Zaporowo				–0.03				

Grayscale indicate the level of significance based on chi-square test—white p < 0.05, light gray p < 0.01, dark gray p < 0.001.

Generally, IADFs type L also showed an increase with precipitation in May and a decrease with precipitation in June and September (Table 6). IADFs type L also occurs more often with temperature in May. The families 2864, 3076, 3078, 2860, and 2514 present more significant relationships with climatic parameters, compared with families 2818, 2817, and 2549, as observed for IADFs type E+. Interestingly, an increase of IADFs type L occurrence with temperature in May was only found in Zaporowo (Table 6).

**Table 6:** **Percent change (positive value indicates increase; negative value indicates decrease) in the odds of the occurrence of IADF type L for each 1-unit increase in monthly climatic data (mean temperature and total precipitation) from May to September, for the period 1990–2006**.

**Family**	**Site**	**Precipitation**					**Temperature**				
		**May**	**June**	**July**	**August**	**September**	**May**	**June**	**July**	**August**	**September**
2818	Młynary	−0.15	0.04			−0.07	−1.58			2.16	
	Zaporowo										
2817	Młynary										
	Zaporowo		−0.07	0.03						−2.15	
2549	Młynary					−0.03					
	Zaporowo	1.04									
2864	Młynary										
	Zaporowo	0.13	−0.11		−0.08	−0.08		−1.98		−2.93	
3076	Młynary	−0.03	1.8	−1.15	3.58	0.95		75.64		134.43	−44.42
	Zaporowo					−0.03					
3078	Młynary			−1.32							
	Zaporowo	0.09	−0.06				1.14	−1.81			1.92
2860	Młynary	−0.13							−1.01		
	Zaporowo	0.6	−0.66		−0.23		15.73	−21.61		−6.48	25.43
2514	Młynary										
	Zaporowo	3.21	−2.06		−0.88	−1.46	36.82			−40.46	

*Grayscale indicate the level of significance based on chi-square test—white p < 0.05, light gray p < 0.01, dark gray p < 0.001*.

Given the limited frequency of the L+ type, no significant relationships were found.

## Discussion

Our results showed that the IADF frequency was largely dependent on the climatic conditions, with IADF type E also showing a significant influence of the site. However, our results did not find a significant effect of the genotype on the formation of the different types of IADFs. Nonetheless, different families appear to show different sensitivities to climatic conditions, reflected in the different correlations between IADFs and climate.

### IADFs and genotypes

The presence of IADFs in the earlywood indicates the capacity of trees to adapt to stress conditions, lowering the risk of xylem cells embolism through a reduction in the lumen area and an increase in cell-wall thickness (Hacke et al., 2001). The presence of density fluctuations within earlywood seems to be influenced by both tree genotype and environmental conditions (Rozenberg et al., 2002). Experiments with clones of Norway spruce showed a strong genetic effect on the occurrence of IADF type E as a reaction to water shortages (Rozenberg et al., 2002). The use of specimens with a high degree of relatedness increases the chances to observe the influence of the genotype on certain traits (Rozenberg and Cahalan, 1997). In our study we have used half-sib families of European larch and this may explain why we could not see a strong effect of the genotype on the occurrence of IADFs type E and E+. This indirectly points out a high level of heritability of the wood density components of the European larch families (Klisz, 2011), has also observed in other tree species (Hylen, 1999; Louzada and Fonseca, 2002; Louzada, 2003). Nonetheless, some of the families of the European larch significantly differ in terms of the frequency of IADFs type E and E+ (Table 3).

The heritability of the latewood density in conifers would predict that the occurrence of IADF type L and L+ was determined by the trees genotypes (Hylen, 1999; Hannrup et al., 2004). However, the frequency of density fluctuations in latewood was similar among the analyzed families of European larch. While some authors point to a higher heritability of earlywood as opposed to latewood (Rozenberg and Cahalan, 1997; Louzada and Fonseca, 2002), others have observed the reverse trend (Goggans, 1964; Hylen, 1999; Hannrup et al., 2004). In our study, the occurrence of IADFs type L was much more dependent on climatic conditions, namely humidity conditions at the end of the growing season. Trees at both experimental sites were characterized by very low frequencies of IADF L+, probably linked to an absence of an autumn growing season that favor the generation of this type of fluctuation (Campelo et al., 2006). Vieira et al. (2009) suggests that this kind of fluctuation is due to the occurrence of climatic conditions unfavorable for the complete maturation of xylem cells.

### IADFs and climate

The frequency of IADFs in annual rings is associated, *inter alia* with the type of climate shaping the course of the processes by which wood cells arise, grow, and differentiate. The low frequency of IADFs in the tree rings of European larch growing in northern Poland, with 9.1 and 11.1% of the annual rings showing IADFs, at the Młynary and Zaporowo sites, respectively, is within the range observed by other authors under temperate and boreal climates (Wimmer et al., 2000; Rigling et al., 2001; Franceschini et al., 2013). Relatively high frequencies of such fluctuations are observed in conifers growing under Mediterranean or Atlantic climate (15–32 and 30–52%, respectively; Campelo et al., 2006; Bogino and Bravo, 2009; Vieira et al., 2009; Rozas et al., 2011). This is probably related with a wider length of the growing season under Mediterranean or Atlantic climate, increasing the probability of environmental variations affecting wood development processes, with the consequent formation of IADFs (Campelo et al., 2015). Nonetheless, the frequency of IADFs in European larch was quite high in specific years, namely in 1998 and 2000, with more than 15% of the trees showing IADFs in Młynary, and in 1997 with more than 40% of the trees showing IADFs at Zaporowo. This could be related with very specific climatic conditions of those years that induced the formation of IADFs in more trees. However, because Młynary and Zaporowo are close by and under similar climatic conditions, and if the formation of IADFs is mainly controlled by climatic conditions, then we would expect that years with a higher frequency of IADFs would be the same among the sites, which was not observed in the mentioned years above. Thus, other site differences (e.g., microclimatic conditions, soil differences) could explain the observed year-lag of IADFs frequency among sites.

Conifers growing in temperate climatic conditions usually present a higher frequency of earlywood IADFs (Rozenberg et al., 2002; Hoffer and Tardif, 2009), while under boreal or alpine climate, latewood fluctuations tend to prevail (accounting for 69–100% of the total; Rigling et al., 2001) as well as under Mediterranean climate (Campelo et al., 2006; Vieira et al., 2009; Nabais et al., 2014). Our results showed that the dominant types of wood density fluctuations in European larch growing in the North of Poland were E+ and L, accounting for 42.6–44.9 and 31.8–35.9% of all reported fluctuations, respectively. This intermediate behavior might be related to the fact that the study sites were located in the transition area from temperate to boreal climate.

In general, all the main types of IADFs found in European larch (type E, E+, and L) showed a significant positive correlation with the precipitation in May. Thus, it seems that higher precipitation levels in May increases the probability of IADFs formation of any type. High water availability during the growing season can increase the division rate of cambial cells (Schuppler et al., 1998; Rossi et al., 2009). Cells must go through a process of growth and maturation, and an increase in the amount of cells means that more cells are available to integrate variations in the environmental conditions, increasing the probability of the occurrence of an IADF (Carvalho et al., 2015). In fact, precipitation in May–July stimulates the growth of larch (Oleksyn and Fritts, 1991; Koprowski, 2012) and as a result wide rings are present. Previous studies have also shown that density fluctuations are more common in wider rings than in narrow ones (Schweingruber, 1980).

The formation of IADFs type E is usually determined by water shortages during the growing season, with the formation of latewood-like cells within earlywood (Rozas et al., 2011). The relation between IADFs type E and climatic conditions among sites and families was quite scattered and thus with no clear pattern. Even so, there was a positive relationship of IADFs type E with the temperature in April and precipitation in May. Although, high precipitation in May does not indicate water stress, high temperatures in April could indicate a potential increase in evapotranspiration and thus water stress. Higher temperatures during the growing season could also increase the respiratory rate, reducing the hexose pool in cambium and xylem (Deslauriers et al., 2016). Hexose is produced to increase cell osmotic potential to generate turgor pressure for cell expansion (Koch, 2004). If there is a lower availability of carbon for growth, particularly for cell enlargement (Deslauriers et al., 2016), then an IADF type E could be formed. Cuny et al. (2014) have also showed that cell enlargement duration contributed to 75% of changes in cell diameter, while the amount of wall material per cell was quite constant. Thus, the thicker walls of an IADF type E do not represent a higher amount of carbon for cell wall thickening but is just a consequence of a lower lumen diameter. Interestingly the site significantly affected the formation of this type of IADF, with a higher frequency in Zaporowo compared to Młynary. A potential environmental factor modeling the reaction of the cambium to drought-associated stress conditions may be the capacity of the soil to retain water (Rozenberg et al., 2002). Soils with a high water storage capacity, mainly determined by soil type and depth, may weaken the impact of shortfalls in precipitation in the first half of the growing season, thereby reducing the frequency of earlywood density fluctuations (Rozenberg et al., 2002). The soil-types were different between the two sites, with a sandy clay soil in Młynary, and a brown earth soil in Zaporowo. It is known that sandy soils retain less water but under water stress conditions plants retrieve water more easily, compared with soils richer in organic matter, like the brown earth soils. Thus, the soil-type might explain the higher frequency of IADFs-type E in Zaporowo, compared with Młynary.

IADFs type E+ can be understood as a smooth transition between early- to latewood, i.e., a smooth decreasing of lumen diameter and increase in cell wall width. Although, there were no significant effects of the family on the formation of IADFs type E+ (*P* = 0.064), the correlations with climatic conditions seems to separate two groups of families, with one group showing positive correlations with precipitation in March and May, and a negative correlation with temperatures in March, and the other group showing positive correlations with precipitation in April and temperatures in April and May. This could indicate a different sensitivity of these two groups of families toward climatic conditions. However, further research is necessary to understand if these dissimilarities are due to genotypic differences.

The mechanism behind the formation of IADFs type L, commonly found in trees from the Mediterranean region, is associated with the cambium reactivation following a summer drought (Vieira et al., 2010; Novak et al., 2013; Nabais et al., 2014). The fact that this kind of fluctuation is prevalent under the circumstances of a Mediterranean climate is related with the bimodal growth pattern present among trees growing in this climatic zone (Cherubini et al., 2003). While the generation of IADF type E is interpreted as a reaction to unfavorable growth conditions in spring, the formation of an IADF-type L is indicative of better conditions for growth at the end of the growing season (Campelo et al., 2007; Nabais et al., 2014). In the case of the European larch growing in Poland, the formation of IADFs type L is generally associated with a positive correlation with precipitation and temperature in May. As observed for IADFs type E+, it appears that some of the families are more sensitive to climatic conditions at the end of the growing season, as indirectly revealed by the occurrence of more significant correlations with climatic conditions and IADFs-type L. Although, there was no significant effect of the site, at least some families growing in Zaporowo showed a negative correlation between IADFs-type L and the precipitation in June and the temperature in August. While the negative correlation with temperature in August makes sense from the point of view of the formation of IADFs type L, the negative correlation with June precipitation is counter-intuitive. To further understand how and when climatic conditions can be imprinted in wood anatomy, studies of xylogenesis of European larch are necessary to see if the timings of the different phases of wood formation differ among families and/or sites.

## Conclusion

The frequency of IADFs under temperate climates is quite low when compared with Mediterranean or Atlantic climates. This is probably related to differences in the length of the growing season. Most of the IADFs found in the European Larch growing under a temperate climate in Northern Poland were of the type E+ and type L. Genetic determination underpinning the adaptation of the radial growth process to anomalies arising cyclically in a temperate climate should mainly concern earlywood and the transition zone with latewood, with a focus on related individuals. Although, we did not find a clear effect of genotype on the frequency of the IADFs, differences occurred in the pairwise comparison of the different families. The influence of a tree's genotype on its predisposition to generate density fluctuations within the annual rings would seem to be dependent on the degree of relatedness. In the European larch, anomalies in the anatomical structure of earlywood and wood of the transition zone are expressed most clearly in clones (i.e., trees that are identical genetically) and weakly among the families has in our study. Besides a general positive correlation of May precipitation and the frequency of IADFs, no clear pattern of the climatic signal of the different types of IADFs could be found. This can be due to the fact that we have used different families weakening a clearer climatic signal. Nonetheless, two groups of families could be distinguished based on their different sensitivity to climatic parameters, although further studies are necessary to confirm this observation.

## Author contributions

MK, MKo, JU, and CN gave a substantial contribution to the conception and design of the study, MK performed x-ray density analyses, MK and JU were in charge of genetic analyses, MKo and JU performed climatic analyses, MK wrote the first draft of the manuscript, MK, MKo, JU, and CN contributed to writing specific sections of the manuscript. All authors contributed to manuscript revision, read, and approved the submitted version.

### Conflict of interest statement

The authors declare that the research was conducted in the absence of any commercial or financial relationships that could be construed as a potential conflict of interest.
